# Structural Variations of Broccoli Polyphenolics and Their Antioxidant Capacity as a Function of Growing Temperature

**DOI:** 10.3390/plants14081186

**Published:** 2025-04-11

**Authors:** Ivana Šola, Daria Gmižić

**Affiliations:** Department of Biology, Faculty of Science, University of Zagreb, Horvatovac 102a, 10000 Zagreb, Croatia

**Keywords:** broccoli, esterified phenolics, free phenolics, glycosylated phenolics, insoluble phenolics, soluble phenolics, temperature

## Abstract

Polyphenolics in plants exist in free, soluble-bound, and insoluble-bound structural forms. The concentration of these structural forms depends on the plant’s developmental stage, tissue type, soil water availability, and food preparation methods. In this study, for the first time, the effects of growth temperature (RT—room temperature—23 °C day/18 °C night, HT—high temperature—38 °C day/33 °C night, LT—low temperature—12 °C day/7 °C night) on variations of polyphenolic structural forms—free, soluble-bound (esterified and glycosylated), and insoluble-bound—in broccoli (*Brassica oleracea* L. convar. *botrytis* (L.) Alef. var. *cymosa* Duch.) microgreens were investigated. Using spectrophotometric, RP-HPLC, and statistical analyses, it was found that the highest amount of total phenolics (TP) in broccoli microgreens was present in the esterified form, regardless of the temperature at which they were grown (63.21 ± 3.49 mg GAE/g dw in RT, 65.55 ± 8.33 mg GAE/g dw in HT, 77.44 ± 7.82 mg GAE/g dw in LT). LT significantly increased the amount of free (from 13.30 ± 2.22 mg GAE/g dw in RT to 18.33 ± 3.85 mg GAE/g dw) and esterified soluble TP (from 63.21 ± 3.49 mg GAE/g dw in RT to 77.44 ± 7.82 mg GAE/g dw), while HT significantly increased the amount of TP glycosylated forms (from 14.85 ± 1.45 mg GAE/g dw in RT to 17.84 ± 1.20 mg GAE/g dw). LT also enhanced free and esterified forms of total flavonoids, tannins, hydroxycinnamic acids, and flavonols. HT, on the other hand, increased glycosylated forms of TP, flavonoids, tannins, hydroxycinnamic acids, flavonols, and phenolic acids, and decreased insoluble-bound tannins. According to the ABTS method, HT induced antioxidant potential of free and glycosylated forms, while LT increased antioxidant capacity of free forms only. According to the FRAP method, LT increased antioxidant potential of free and esterified polyphenolic forms. Also, based on ABTS and FRAP assays, esterified polyphenolics showed significantly higher antioxidant capacity than any other form. Principal component analysis showed that structural form had a greater impact than temperature. Hierarchical clustering showed that RT-, HT- and LT-broccoli microgreens were most similar in their glycosylated polyphenolics, but differed the most in esterified forms, which were also the most distinct overall. In conclusion, HT and LT induced specific shifts in the structural forms of broccoli polyphenolics and their antioxidant capacity. Based on the results, we recommend applying LT to increase the amount of free and esterified polyphenolics in broccoli microgreens, while HT may be used to enhance glycosylated forms.

## 1. Introduction

Polyphenolic compounds are present in plants in three different forms: free, soluble-bound, and insoluble-bound [1]. Free phenolics can be extracted using solvents and are readily absorbed in the small intestine. Soluble-bound phenolics, which are either esterified or glycosylated, are loosely bound to low-molecular-mass compounds such as sugars or fatty acids. They are also soluble in aqueous/organic solvents and can be absorbed in the small intestine. Insoluble-bound phenolics are linked to structural cell wall components such as pectin, cellulose, hemicellulose, and lignin, making them less metabolically available and more difficult to extract [2]. These compounds are not absorbed in the small intestine but can be released in the colon by microbial activity [3]. Insoluble-bound phenolics contribute to 20–60% of the total phenolic compounds in food matrices and might even reach 99% in cereal brans [4]. To date, most research has focused on soluble free forms [5], with less attention given to bound forms, and the least amount of data is available on insoluble-bound polyphenolics [4,6]. This is likely due to differences in the complexity of extraction processes, and the high cost of glycosylated phenolic standards, which are more expensive than their aglycone counterparts. For example, in our previous studies, we predominantly focused on aglycone forms, which we released using acid hydrolysis [7], while glycosylated forms of flavonoids were analyzed less frequently [8] due to the higher cost of standards. Additionally, research on soluble phenolics often yields faster results due to simpler extraction and analysis processes. This makes them more appealing for initial screening studies, particularly when time or resources are limited. However, once ingested, bound polyphenolics can be hydrolyzed by enzymes and microorganisms in the gastrointestinal system, converting them into free forms that can be absorbed [6]. The profiles of insoluble bound phenolic compounds in different plant foods are discussed in detail in the review of Rocchetti et al. [9].

To date, the majority of research has focused on the distribution of different polyphenolic structural forms in various plant organs and tissues, as well as their correlations with antioxidant potential [4,10,11,12,13,14]. Shahidi and Hossain [4] reported that in general, fruits and vegetables contain higher amounts of soluble free or conjugated phenolics than insoluble-bound phenolics, whereas grains have a higher proportion of insoluble-bound phenolics. They also explained that bound phenolics are primarily found in plant protective tissues like the seed coat, pericarp, and hull, but they are also present in nutritional tissues, including the germ, epicotyl, hypocotyl, radicle, and endosperm. Acosta-Estrada et al. [15] reviewed the proportions of insoluble-bound polyphenolics relative to total polyphenolics of different food sources, showing that apple contained only 6.5% [16], while brown rice had 88% [17], and Maverick pinto bean even as much as 98% [18]. Additionally, studies have investigated the influence of different extraction solvents [12] and food preparation methods on the concentration of soluble and bound polyphenolics [19,20,21,22,23,24]. Harbaum et al. [10] found that the concentrations of cell wall-bound phenolic compounds were higher in the leaf blade than in the leaf stalk of pak choi (*Brassica campestris* L. ssp. *chinensis* var. *communis*) cultivars. The effect of germination on the dynamics of free and bound polyphenolics in plants has also been documented [25].

Regarding the effect of environmental conditions on the profile of soluble and insoluble polyphenolics, de Camargo et al. [26] investigated the impact of drought on different flavonoid forms in chickpeas (*Cicer arietinum* L.). They found that drought preferentially modulates the biosynthesis of free rather than esterified biochanin A. Similarly, Martini et al. [27] examined the effect of crop year and growing area on the content of free, conjugated, and bound polyphenolics in durum wheat (*Triticum turgidum* L. var. *durum*). Variations in the profiles of free and conjugated compounds in quinoa (*Chenopodium quinoa* Willd.) seeds under different environmental conditions have also been documented [28]. As for cruciferous vegetables, research has primarily focused on the effects of environmental factors on soluble compounds [29,30,31,32,33,34,35,36].

Since different structural forms of polyphenolics exhibit distinct biological activities and environmental conditions clearly influence their formation, the aim of this study was to determine the effect of different growing temperatures (RT, HT, and LT) on the dynamics of polyphenolic structural forms in broccoli microgreens and their antioxidant potential. Our hypothesis was that HT and LT would alter the ratio of different structural forms in broccoli compared to RT as a control, and that HT and LT would have distinct effects on polyphenolic structural forms. To test this hypothesis, we (a) grew broccoli microgreens under different temperature regimes (RT, HT, and LT), (b) extracted different structural forms of polyphenolics from the microgreens, (c) spectrophotometrically measured the content of structural forms of different polyphenolic groups, (d) separated, identified, and quantified ferulic and sinapic acid in each of the structural forms extracted using high-performance liquid chromatography (HPLC), (e) determined the antioxidant capacity of different polyphenolic structural forms, (f) statistically analyzed all the data using one-way analysis of variance (ANOVA), principal component analysis (PCA), hierarchical clustering (HC), and Pearson’s correlation of coefficients.

## 2. Results and Discussion

The bioactivity of polyphenolics is highly dependent on their structure [37]. Since polyphenolics contain hydroxyl and carboxyl groups, they can conjugate with other compounds, such as sugars and organic acids [38], forming glycosidic or esterified forms. Therefore, in addition to their free forms, polyphenolics in plants also exist in bound forms, both soluble and insoluble. Soluble polyphenolics, mainly stored in the vacuole, play a role in plant-environment interactions, while insoluble polyphenolics, bound to cell wall components, contribute to maintaining plant structural integrity [39]. Free, soluble, and insoluble polyphenolics have different health effects in humans. Specifically, the consumption of insoluble-bound forms has been linked to protective effects against colon cancer, while free and soluble polyphenolics inhibit LDL cholesterol and liposome oxidation [40]. The structural properties of polyphenols influence intestinal absorption through specific transporters in the small intestine or microbial enzymatic activity in the colon [10]. Different structural forms exhibit varying stability and bioavailability [41]. If temperature fluctuations favor forms with higher or lower stability/bioavailability, this can significantly impact their functional benefits. Understanding how temperature affects structural variations allows for more precise predictions of climate change’s impact on the nutritional and health value of plants. Additionally, structural properties determine how polyphenolics interact with proteins, lipids, and other biomolecules, influencing their solubility, transport, and synergistic or antagonistic effects. Temperature-induced shifts may alter these interactions, affecting both plant physiology and human health benefits. By examining structural transformations, we can get deeper insights into how environmental factors modulate polyphenolic composition and function, ultimately shaping plant resilience and dietary health potential.

### 2.1. The Effect of High and Low Growth Temperature on Free and Bound Total Phenolics in Broccoli

**Figure 1:**
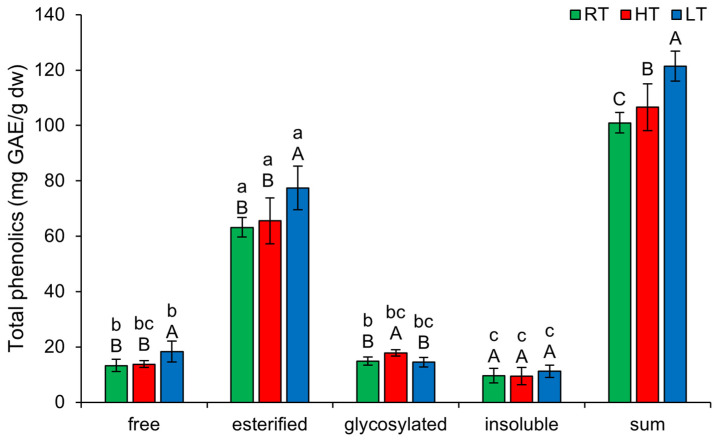
The effect of HT and LT on different structural forms of total phenolics in broccoli microgreens. RT = room temperature, HT = high temperature, LT = low temperature, GAE = gallic acid equivalents. Different capital letters denote significant differences between temperature treatments within the same structural form, and different small letters denote significant differences between structural forms within the same temperature treatment.

The highest amount of TP in broccoli microgreens was present in the esterified form, regardless of the temperature at which they were grown (63.21 ± 3.49 mg GAE/g dw in RT, 65.55 ± 8.33 mg GAE/g dw in HT, 77.44 ± 7.82 mg GAE/g dw in LT) (Figure 1). This implies that the biosynthesis and storage of esterified phenolics are tightly regulated by the plant’s genetic and enzymatic mechanisms, rather than being highly influenced by external temperature changes. This stability could be beneficial for maintaining the nutritional and antioxidant properties of broccoli microgreens under varying environmental conditions. The lowest amount was present in the insoluble-bound form (9.60 ± 2.62 mg GAE/g dw in RT, 9.42 ± 3.17 mg GAE/g dw in HT, 11.18 ± 2.27 mg GAE/g dw in LT). This accounted for about 10% of the total phenolics measured and is quite a higher proportion than less than 2% recorded in leaves of mature Pak Choi (*Brassica campestris* L. ssp. *chinensis* var. *communis*) [10]. Insoluble-bound polyphenolics are typically covalently linked to cell wall components such as lignin, cellulose, or hemicellulose [39] and this makes them less mobile than soluble polyphenolics. The primary role of bound forms is structural, they strengthen the cell wall and contribute to the boundary of plants toward pathogens [39]. The sum of all structural forms of TP increased substantially under both temperature stresses, but LT showed a significantly higher impact than HT.

LT significantly increased the amount of free (from 13.30 ± 2.22 mg GAE/g dw in RT to 18.33 ± 3.85 mg GAE/g dw) and esterified soluble TP (from 63.21 ± 3.49 mg GAE/g dw in RT to 77.44 ± 7.82 mg GAE/g dw), while HT significantly increased the amount of TP glycosylated forms (from 14.85 ± 1.45 mg GAE/g dw in RT to 17.84 ± 1.20 mg GAE/g dw). Insoluble-bound TP was not affected by any of the temperature conditions. Free phenolics act as antioxidants rapidly, whereas esterified forms must first be hydrolyzed into their free forms to become active. Their solubility enhances their ability to circulate and provide localized protection within cells. Glycosylation reduces reactivity while increasing the water solubility of phenolics [42]. These forms are better protected from degradation or oxidation and can therefore be stored for long-term defense or metabolic use [43]. Additionally, glycosylated phenolics may help retain water molecules within their structure, thereby preserving cell turgor [44].

### 2.2. The Effect of High and Low Growth Temperature on Free and Bound Total Flavonoids in Broccoli

**Figure 2:**
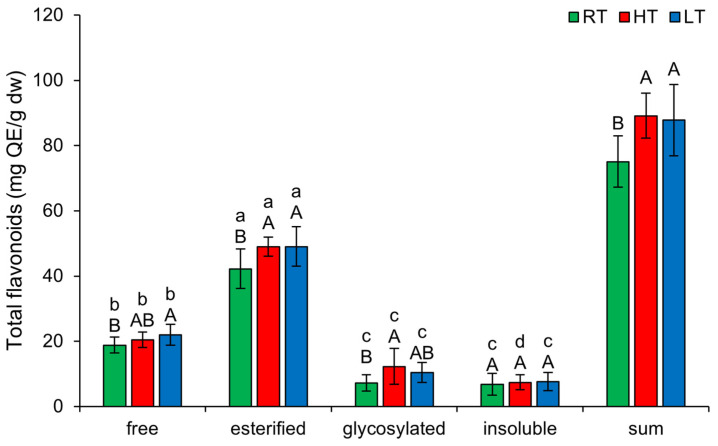
The effect of HT and LT on different structural forms of total flavonoids in broccoli microgreens. RT = room temperature, HT = high temperature, LT = low temperature, QE = quercetin equivalents. Different capital letters denote significant differences between temperature treatments within the same structural form, and different small letters denote significant differences between structural forms within the same temperature treatment.

Under each growing temperature, the order of TF forms from the highest to the lowest concentration was as follows: esterified, free, glycosylated, insoluble-bound (Figure 2). LT markedly increased the amount of free (from 18.80 ± 2.44 mg QE/g dw to 22.00 ± 3.24 mg QE/g dw) and esterified TF (from 42.23 ± 6.02 mg QE/g dw to 49.01 ± 6.05 mg QE/g dw) in broccoli. HT, on the other hand, increased the amount of esterified (from 42.23 ± 6.02 mg QE/g dw to 49.02 ± 2.95 mg QE/g dw) and glycosylated TF (from 7.27 ± 2.51 mg QE/g dw to 12.27 ± 5.53 mg QE/g dw) forms. A possible reason for the glycosylation of flavonoids under HT conditions might be the fact that glycosylated flavonoids are more resistant to heat treatment than aglycon flavonoids [45]. Both HT and LT markedly enhanced the sum of structural forms of TF.

### 2.3. The Effect of High and Low Growth Temperature on Free and Bound Total Tannins in Broccoli

**Figure 3:**
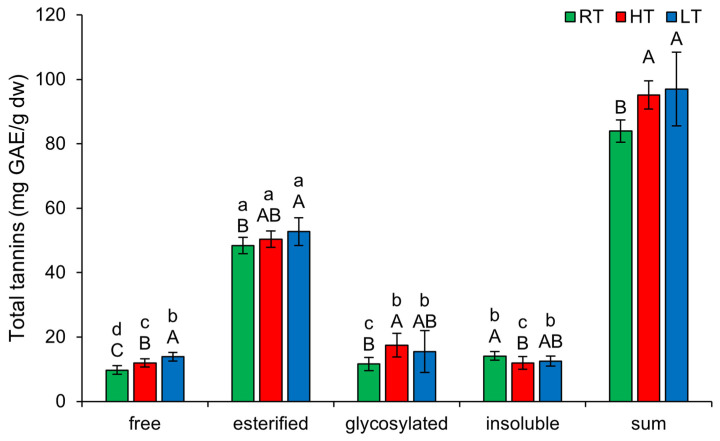
The effect of HT and LT on different structural forms of total tannins in broccoli microgreens. RT = room temperature, HT = high temperature, LT = low temperature, GAE = gallic acid equivalents. Different capital letters denote significant differences between temperature treatments within the same structural form, and different small letters denote significant differences between structural forms within the same temperature treatment.

The biological activities of tannins are based on their three general characteristics—the possibility to complex metal ions, antioxidant and radical scavenging activities, and ability to complex with proteins and polysaccharides [46]. As shown in Figure 3, similar to TP, the highest amount of TT in broccoli microgreens was present in the esterified form, regardless of the temperature at which they were grown (48.41 ± 2.48 mg GAE/g dw in RT, 50.42 ± 2.57 mg GAE/g dw in HT, 52.75 ± 4.33 mg GAE/g dw in LT). Both HT and LT increased free TT, with LT showing a more pronounced effect. Esterified TT increased only under LT, while glycosylated TT increased under HT. HT also decreased insoluble-bound TT in broccoli. Under extreme temperature conditions, tannins may bind to proteins and polysaccharides to enhance their stability and resistance to degradation. An increase in tannin content under HT conditions has previously been observed in the branchlets of *Casuarina equisetifolia* [2]. Similarly, leaves of big trefoil (*Lotus uliginosus* Schkuhr) grown under HT also had a higher amount of condensed tannins [47]. Under dry and warm conditions, *Quercus rubra* produced larger amounts of tannins, which were less polymerized [48]. HT also led to an increase in condensed tannins in grapes [49]. Regarding LT, recent studies have shown that in *Juglans regia* microclones exposed to cold stress, the concentration of condensed tannins increased [50]. Overall, the total content of all structural forms of TT increased substantially under both temperature stress conditions.

### 2.4. The Effect of High and Low Growth Temperature on Free and Bound Total Hydroxycinnamic Acids in Broccoli

**Figure 4:**
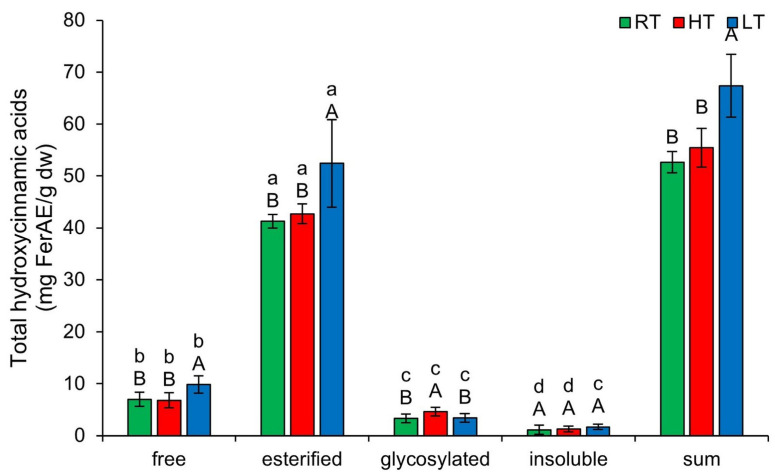
The effect of HT and LT on different structural forms of total hydroxycinnamic acids in broccoli microgreens. RT = room temperature, HT = high temperature, LT = low temperature, FerAE = ferulic acid equivalents. Different capital letters denote significant differences between temperature treatments within the same structural form, and different small letters denote significant differences between structural forms within the same temperature treatment.

Hydroxycinnamic acids are a group of polyphenolic compounds that plants use to enhance stress tolerance [51]. Their mechanisms of action are not yet fully understood; however, it is known that they function through multiple pathways, including antioxidant activity, gene regulation, hormone signaling, and the modulation of phenolic biosynthesis pathways [51]. Additionally, they serve as precursors for monolignols in lignin biosynthesis, thereby helping to maintain cell wall structure. Figure 4 shows the effect of growing temperature on different forms of THCA. LT induced free (from 6.97 ± 1.37 mg FerAE/g dw in RT to 9.86 ± 1.66 mg FerAE/g dw) and esterified (from 41.24 ± 1.81 mg FerAE/g dw in RT to 52.43 ± 8.44 mg FerAE/g dw), while HT increased glycosylated forms (from 3.31 ± 0.82 mg FerAE/g dw in RT to 4.61 ± 0.85 mg FerAE/g dw). Increased esterification of THCA under LT may reduce cell wall permeability [52], thereby contributing to the plant’s physical defenses. Recent studies have shown that UDP-glycosyltransferases (UGTs) in rice (*Oryza sativa* L.) and *Arabidopsis* are upregulated by HT [53]. Therefore, we suggest that the increase in glycosylated THCA forms in broccoli microgreens may result from increased UGT activity. At each growing temperature, the relative concentrations of THCA form, from highest to lowest, following this order: esterified, free, glycosylated, insoluble-bound. The total content of all structural THCA forms increased substantially only under LT stress.

### 2.5. The Effect of High and Low Growth Temperature on Free and Bound Total Flavonols in Broccoli

**Figure 5:**
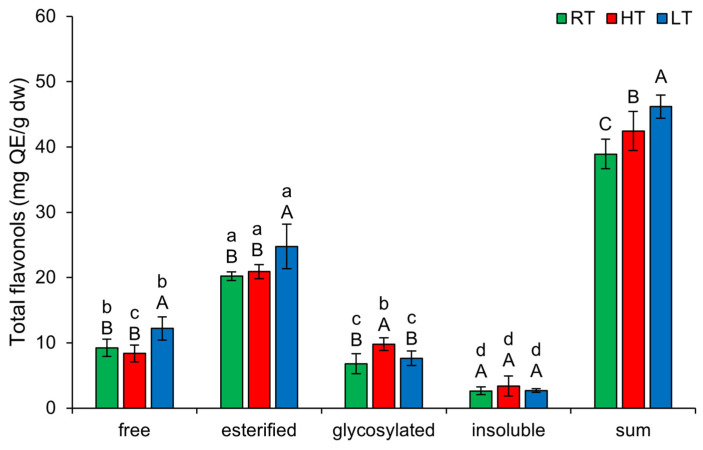
The effect of HT and LT on different structural forms of total flavonols in broccoli microgreens. RT = room temperature, HT = high temperature, LT = low temperature, QE = quercetin equivalents. Different capital letters denote significant differences between temperature treatments within the same structural form, and different small letters denote significant differences between structural forms within the same temperature treatment.

The highest amount of TFLO in broccoli microgreens was present in esterified forms (Figure 5). LT significantly induced free (from 9.25 ± 1.31 mg QE/g dw in RT to 12.19 ± 1.76 mg QE/g dw) and esterified TFLO (from 20.20 ± 0.63 mg QE/g dw in RT to 24.77 ± 3.43 mg QE/g dw). HT growth increased glycosylated forms of TFLO (from 6.82 ± 1.53 mg QE/g dw in RT to 9.77 ± 0.97 mg QE/g dw) in broccoli. This suggests that under HT, broccoli tends to increase the solubility, storage, and stability of flavonols [54]. The total content of all structural TFLO forms increased substantially under both temperature stress conditions, with LT showing a more significant effect than HT.

### 2.6. The Effect of High and Low Growth Temperature on Free and Bound Total Phenolic Acids in Broccoli

**Figure 6:**
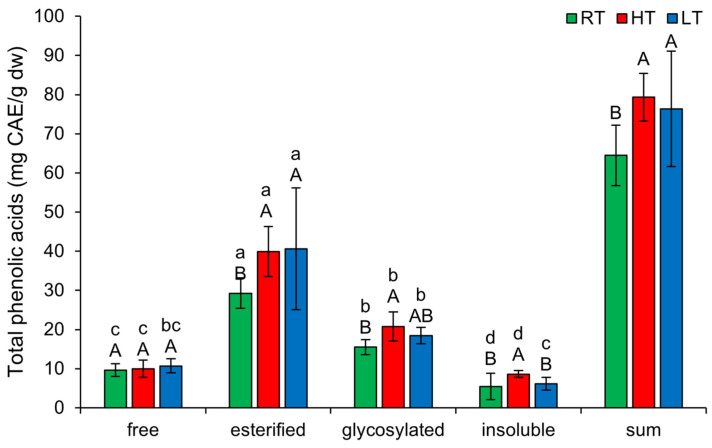
The effect of HT and LT on different structural forms of total phenolic acids in broccoli microgreens. RT = room temperature, HT = high temperature, LT = low temperature, CAE = caffeic acid equivalents. Different capital letters denote significant differences between temperature treatments within the same structural form, and different small letters denote significant differences between structural forms within the same temperature treatment.

Under each growing temperature condition, the TPA forms were ranked in descending order of concentration as follows: esterified, glycosylated, free, insoluble-bound (Figure 6). HT induced the amount of esterified (from 29.29 ± 3.89 mg CAE/g dw in RT to 39.94 ± 6.37 mg CAE/g dw), glycosylated (from 15.55 ± 1.92 mg CAE/g dw in RT to 20.82 ± 3.73 mg CAE/g dw), and insoluble-bound TPA (from 5.42 ± 3.38 mg CAE/g dw in RT to 8.61 ± 0.86 mg CAE/g dw). Since insoluble-bound phenolics can resist enzymatic hydrolysis and absorption in the colon, they may function as prebiotics [55]. Therefore, HT stress could potentially enhance the prebiotic properties of broccoli microgreens. LT increased only the esterified forms (from 29.29 ± 3.89 mg CAE/g dw in RT to 40.64 ± 15.55 mg CAE/g dw). The fact that both temperature stresses enhanced the esterification of phenolic acids suggests potential reinforcement of the cell wall through the formation of ester bonds with hemicellulose or pectin [56]. The total content of all structural TPA forms increased substantially under both temperature stress conditions.

We also examined the proportion of each structural form within each polyphenolic group (Figure S1). The highest proportion of esterified forms was recorded among THCA, which aligns with the fact that hydroxycinnamic acids are typically esterified with quinic and tartaric acids or various carbohydrates [57]. The highest proportion of free forms was found in TF, while glycosylated forms were most prevalent in TPA, and insoluble-bound forms dominated in TT. The glycosylation of phenolic acids may reduce their reactivity and enhance their stability [58,59]. Tannins are generally highly bound to cell wall polysaccharides [60], which may explain why they were predominant in the insoluble-bound fraction. According to Shahidi and Hossain [4], the insoluble-bound fraction has a less diverse phenolic profile than the free phenolic fraction, with phenolic acids and condensed tannins being the dominant components.

In general, glycosylation increases the water solubility of hydrophobic metabolites, facilitating their transport through cell membranes and distribution [43]. It also reduces the volatility of small compounds, which may help prevent the loss of volatile compounds under HT conditions. Additionally, the attachment of sugar moieties to small metabolites increases their size and enhances thermal stability by raising their melting point, aiding plants in coping with HT stress. Furthermore, Su et al. [61] found that UDP-glucosyltransferase rUGT73A17 in tea (*Camellia sinensis*) plants exhibited higher expression levels and activity at HT, which aligns with our findings. Regarding the increased esterification under LT conditions, we hypothesize that polyphenolics contribute to LT resilience by further crosslinking lignin and carbohydrates in the cell wall [62]. Under LT, lignin accumulation is enhanced [63], which helps mitigate intracellular damage. Thus, esterified polyphenolics predominantly serve structural or protective roles under LT conditions, whereas glycosylated forms support plant tolerance to HT. Additionally, glycosylated phenolics may act as long-term reserves, sustaining the plant through extended periods of stress. These adaptations reflect the plant’s strategic response to various stressors, ensuring survival and maintaining metabolic balance under different environmental conditions.

### 2.7. The Effect of High and Low Growth Temperature on Free and Bound Individual Polyphenolics in Broccoli

We also performed RP-HPLC analysis of individual hydroxycinnamic acids, ferulic, and sinapic acids. Both were found to be present in the highest concentrations in esterified forms, followed by free and insoluble-bound forms, while glycosylated forms were present in the lowest concentrations (Table 1). Among the structural forms of ferulic acid in the RT group, esterified forms were the most abundant, followed by the other forms, which did not markedly differ from each other. In the HT broccoli group, esterified forms of ferulic acid were also considerably more prevalent, followed by the free form, with glycosylated and bound forms present in the lowest concentrations, and no differences between these two. In the LT group, esterified forms were predominant as well, followed by the other forms, which showed no differences among them. For sinapic acid in the RT group, esterified forms were also the most abundant, followed by the free form, while glycosylated and bound forms had the lowest concentrations. In the HT group, as with ferulic acid, esterified forms of sinapic acid were significantly more prevalent, followed by the free form, and glycosylated and bound forms were present in the lowest concentrations with no differences between them. In the LT group of broccoli, sinapic acid was predominantly represented in esterified and free forms, followed by glycosylated and bound forms.

Compared to the RT group, free ferulic acid was significantly increased by HT (135.47%), while both free and esterified sinapic acid was notably decreased by LT (57.50%). This suggests that ferulic acid plays a role in the adjustment of broccoli microgreens to HT, while sinapic acid acts as an intermediary in the response to LT. We observed similar results in our previous studies on microgreens grown under HT conditions [29] and in young broccoli plants treated with hot water [30]. Consistent with our results, Cheng et al. [64] reported that pre-treatment with 0.6 mM ferulic acid for 1 day helped blueberry (*Vaccinium corymbosum*) seedlings cope with heat stress by boosting antioxidant enzyme activity, proline accumulation, and soluble sugar levels. Alhaithloul et al. [65] also observed a significant increase in ferulic acid in tomato seedlings (*Solanum lycopersicum* L.) treated with heat shock (45 °C and 50 °C). Moreover, Khan et al. [66] recently published a review on the therapeutic potential of ferulic acid to enhance plant resistance to abiotic stresses and support sustainable crop production. As for free sinapic acid, the results we observed were similar to those reported by Kaplan et al. [67], who studied *Arabidopsis* treated with cold shock stress. In our previous work, where we treated the extracts with 1.2 M HCl instead of 6 M HCl used in this study to release free compounds, we found that LT stress also increased sinapic acid in broccoli microgreens [31]. In winter oilseed rape leaves (*Brassica napus* L. var. *oleifera* L. cv. Jantar) subjected to cold and then freezing treatments, soluble ferulic acid also increased [68].

### 2.8. The Effect of High and Low Growth Temperature on Antioxidant Capacity of Free and Bound Polyphenolics in Broccoli

According to the ABTS method, HT increased the antioxidant potential of free and glycosylated forms, while LT enhanced the antioxidant capacity of free forms only (Table 2). The DPPH method revealed a decrease in the antioxidant capacity of glycosylated polyphenolic forms in broccoli grown under HT conditions. Based on the FRAP method, LT increased the antioxidant potential of free and esterified polyphenolic forms. Additionally, ABTS and FRAP assays showed that esterified polyphenolics exhibited significantly higher antioxidant capacity than any other polyphenolic form. These results suggest that structural variations in polyphenolics do not have a unidirectional effect but instead exert a highly matrix-dependent influence on antioxidant capacity. We assume that pH conditions and interactions with other biomolecules may contribute to variations in antioxidant capacity. For comparison, Huilan et al. [69] reported that free phenolic/flavonoid contents in six buckwheat (*Fagopyrum esculentum*) varieties exhibited higher antioxidant activities (DPPH, ABTS, OH^•^, and FRAP) than bound phenolics. However, in Kainth (*Pyrus pashia*) fruit, bound polyphenolics demonstrated higher antioxidant capacity than free and esterified forms [70]. Similarly, significantly stronger antioxidant activities were observed for free phenolics compared to esterified and insoluble-bound phenolics of *Lonicera japonica* and *L. macranthoides* [71].

Figure S2 illustrates the intensity and direction of changes in different structural forms of polyphenolic groups in broccoli, along with their antioxidant capacity. It is evident that most free polyphenolic groups, except TPA, substantially increased when broccoli was grown under LT conditions (Figure S2A). Meanwhile, under HT stress, among the free groups, only TT was notably enhanced. This suggests that LT conditions may trigger metabolic adjustments in broccoli, leading to the upregulation of specific biosynthetic pathways involved in polyphenolic compound production. Additionally, the highest level of change among free polyphenolic groups was observed for total tannins under LT (42.16%). The dynamics of esterified polyphenolic groups under HT and LT are shown in Figure S2B. LT markedly increased all esterified polyphenolic groups, with TPA exhibiting the most pronounced change (38.72%). Under HT conditions, esterified TPA and TF were also elevated. Glycosylated forms of polyphenolic groups in broccoli exhibited more pronounced changes under HT than LT (Figure S2C). HT increased all polyphenolic groups in their glycosylated forms, with the highest change observed in total flavonoids (68.70%). Interestingly, LT did not considerably affect any of the glycosylated polyphenolic groups. Insoluble-bound polyphenolics were the most specifically affected by HT and LT (Figure S2D). Insoluble-bound tannins were markedly decreased by HT, while HT increased insoluble-bound total phenolic acids by 59.03%. In contrast, LT led to an increase in insoluble-bound total hydroxycinnamic acids. The sum of all structural forms within each polyphenolic group was markedly increased by LT, with total hydroxycinnamic acids showing the highest change (25.87%). Under HT conditions, the sum of structural forms of each polyphenolic group, except total hydroxycinnamic acids, also notably increased. Among the identified individual compounds, free ferulic acid was notably increased by HT, while free sinapic acid was enhanced by LT. Bound soluble and insoluble forms of these acids in broccoli microgreens were not significantly affected by HT and LT. Regarding antioxidant potential, LT increased the capacity of free (ABTS and FRAP) and esterified phenolics (FRAP), while HT increased the capacity of free and glycosylated forms (ABTS).

### 2.9. Statistical Analyses

#### 2.9.1. Pearson’s Correlations

Pearson’s correlation coefficients indicate whether a linear relationship exists between two variables, as well as the strength and direction of this relationship [72]. Given the significant changes in the structural forms of polyphenolics in broccoli under different temperature conditions, we conducted a correlation analysis to explore potential relationships between these structural forms and the antioxidant potential of broccoli. The interpretation of correlation levels was based on the table provided by Meghanathan [73].

As shown in Table 3, within the fraction containing free polyphenolics, total tannins exhibited the highest correlation coefficient with total flavonoids. Total flavonols showed the strongest correlation with sinapic acid concentration. Antioxidant capacity, as measured by the ABTS assay, displayed the highest correlation coefficient (*r* = 1.00) with total tannins and flavonoids, as well as a very strong correlation (*r* = 0.94) with total phenolic acids. This indicates that the antioxidant capacity (ABTS) was strongly influenced by free forms of tannins, flavonoids, and phenolic acids. The DPPH assay also showed the highest correlation with total tannins (*r* = 1.00), along with very strong correlations with total phenolic acids (*r* = 0.97) and total phenolics (*r* = 0.91). This suggests that free forms of total tannins play a key role in scavenging free radicals. The FRAP assay exhibited the highest correlation (*r* = 1.00) with total hydroxycinnamic acids and very strong correlations with total phenolics, flavonols, and sinapic acid (all *r* = 0.99), as well as total phenolic acids (*r* = 0.94). In conclusion, within the fraction of free components, total tannins and flavonoids appear to be the dominant contributors to antioxidant activity, as demonstrated by both the ABTS and DPPH assays. Meanwhile, free hydroxycinnamic acids, flavonols, and sinapic acid were more effective in the FRAP assay, highlighting their strong reducing potential.

In the fraction containing esterified polyphenolics, a perfect positive correlation (*r* = 1.00) was recorded between antioxidant capacity measured by FRAP and total hydroxycinnamic acids. This suggests that hydroxycinnamic acids are the primary contributors to the reducing power in this fraction. One possible explanation is that these compounds contain ortho- or para-hydroxyl groups on their aromatic rings, which are highly efficient in electron donation, and esterification may enhance electron delocalization. Additionally, esterified hydroxycinnamic acids are often bound to cell wall components or other molecules, and esterification may improve their solubility and availability, thereby enhancing their interactions with the FRAP reagent. Very strong correlations were also observed between FRAP and total phenolics and flavonols (both *r* = 0.99), as well as total tannins (*r* = 0.90).

In the fraction containing glycosylated forms, the ABTS assay showed a perfect correlation with total flavonols (*r* = 1.00), and a very strong correlation with total phenolic acids, flavonoids, hydroxycinnamic acids, and tannins (*r* = 0.98, *r* = 0.96, *r* = 0.95, *r* = 0.94, respectively). Similarly, the FRAP assay exhibited a perfect correlation with total hydroxycinnamic acids (*r* = 1.00), and a very strong correlation with total phenolics (*r* = 0.99), flavonols (*r* = 0.97), and ferulic acid (*r* = 0.90).

In the fraction containing insoluble-bound polyphenolics, ABTS showed a very strong correlation with total phenolics (*r* = 0.96). DPPH exhibited a very strong positive correlation with total flavonoids (*r* = 0.89) and phenolic acids (*r* = 0.83). FRAP showed a perfect correlation with total flavonoids (*r* = 1.00) and a very strong correlation with total hydroxycinnamic acids (*r* = 0.99). Additionally, we observed absolute negative correlations in this fraction, specifically between DPPH and sinapic acid (*r* = −1.00) and between FRAP and ferulic acid (*r* = −1.00). This suggests that the higher the proportion of insoluble-bound sinapic and ferulic acid, the lower the recorded antioxidant capacity. These findings indicate that soluble forms of these two hydroxycinnamic acids are crucial for the antioxidant potential of broccoli microgreens.

In general, FRAP results showed a perfect positive correlation with hydroxycinnamic acids in every fraction type. This suggests that, in addition to their hydrogen- or electron-donating ability and the stability of the resulting phenoxyl radicals, these acids also reduced ferric ions more effectively than the other phenolics studied.

When we summed up all the extracted fractions, total flavonols were absolutely (*r* = 1.00) correlated with ABTS and FRAP. DPPH was very strongly correlated with each of the polyphenolic groups, ABTS was very strongly correlated with total flavonoids, tannins, and hydroxycinnamic acids, while FRAP was very strongly correlated with total phenolics, tannins, and hydroxycinnamic acids.

Regarding the relationships between antioxidant assay results, in the fraction containing free structural forms, DPPH antioxidant capacity was perfectly correlated with ABTS antioxidant capacity, and showed a very strong correlation with the FRAP result (*r* = 0.84). In the fraction with esterified forms, a very strong correlation was observed between ABTS and DPPH (*r* = 0.81) as well as between ABTS and FRAP (*r* = 0.80). For the fraction containing glycosylated polyphenolics, a very strong correlation was detected between ABTS and FRAP results (*r* = 0.94). However, in this fraction, DPPH was not positively correlated with ABTS and FRAP results, likely because these forms interact differently in these assays. Specifically, glycosylation affects the solubility of polyphenolics, which in turn may alter their reactivity under different assay conditions. In the fraction containing insoluble-bound polyphenolics, a very strong correlation was recorded between FRAP and DPPH results (*r* = 0.86).

#### 2.9.2. Principal Component Analysis

**Figure 7:**
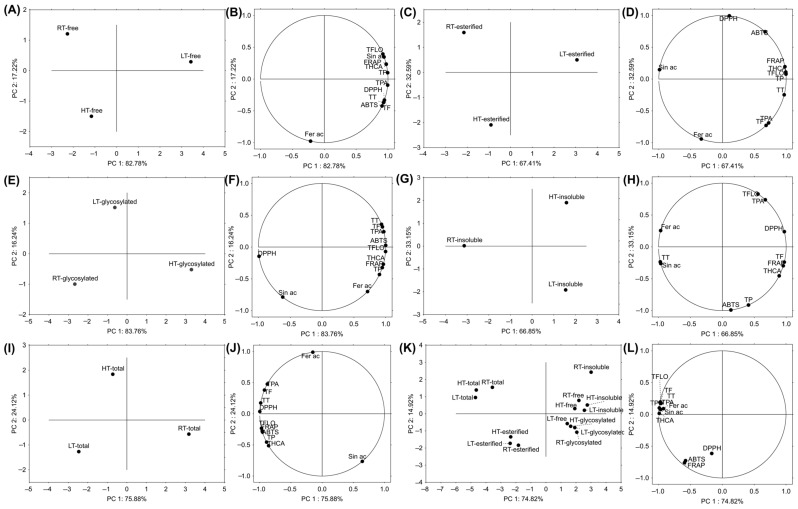
The principal component analysis showing the relation among RT-, LT- and HT-grown broccoli microgreens (**A**) free structural forms and (**B**) grouping of the analyzed variables; (**C**) esterified structural forms and (**D**) grouping of the analyzed variables; (**E**) glycosylated structural forms and (**F**) grouping of the analyzed variables; (**G**) insoluble-bound forms and (**H**) grouping of the analyzed variables; (**I**) sum of all the structural forms and (**J**) grouping of the analyzed variables; (**K**) all the analyzed structural forms and (**L**) grouping of the analyzed variables. ABTS = 2,2′-azino-bis(3-ethylbenzothiazoline-6-sulfonic acid), DPPH = 2,2-diphenyl-1-picrylhydrazyl, Fer ac = ferulic acid, FRAP = ferric ion reducing antioxidant power, HT = high temperature, LT = low temperature, RT = room temperature, Sin ac = sinapic acid, TF = total flavonoids, TFLO = total flavonols, THCA = total hydroxycinnamic acids, TP = total phenolics, TPA = total phenolic acids, TT = total tannins.

Principal Component Analysis (PCA) is a statistical technique used to simplify big datasets while preserving as much variability as possible [74]. It uses algorithms to transform the original variables into a smaller number of new variables, called principal components (PC), which are uncorrelated and ordered by the amount of variance they explain in the data. In this study, we aimed to test how HT and LT affected different structural forms of polyphenolics in broccoli and to reveal how these forms would cluster in relation to growth temperature. Based on the free structural forms, LT-grown broccoli was separated from those grown under RT and HT conditions (Figure 7A). The main variables contributing to this clear separation were total flavonols, hydroxycinnamic acids, phenolics, sinapic acid, and antioxidant potential (FRAP) (Figure 7B). Esterified forms also separated LT-grown broccoli from RT and HT groups (Figure 7C), with the main contributing variables being total hydroxycinnamic acids, flavonols, phenolics, and antioxidant capacity (FRAP, ABTS, and DPPH) (Figure 7D). Glycosylated structural forms separated the HT group of broccoli from the RT and LT groups (Figure 7E). The variables that contributed to this separation were total hydroxycinnamic acids, phenolics, flavonols, ferulic acid, and antioxidant capacity (FRAP) (Figure 7F). We hypothesize that under HT, glycosyltransferases are enhanced [75] leading to a higher concentration of glycosylated phenolics. Insoluble-bound phenolics separated RT broccoli from those grown under temperature stress (Figure 7G). The variables that contributed the most to this separation were total tannins, ferulic, and sinapic acid (Figure 7H). As shown earlier, HT notably decreased total tannins (Figure 7), while both temperature stresses decreased ferulic and sinapic acid concentrations (Table 1). We hypothesize that under temperature stress, these components shift from insoluble-bound forms, which primarily serve to reinforce the cell wall [56], to free and/or soluble-conjugated forms to help the plant defend against oxidative damage more immediately. Since we have already observed the relevance of sinapic and ferulic acids in broccoli’s response to temperature stress [29,31], such a result was expected. Based on the sum of different structural forms, RT-grown broccoli was separated from those grown under LT and HT (Figure 7I). The most contributing variable was sinapic acid (Figure 7J). When we analyzed all the samples together (from different temperatures and structural forms), two distinct clusters emerged: one for esterified structural forms and another for the sum of all structural forms (Figure 7K). Among insoluble-bound polyphenolics, the RT sample was notably distinguished from the LT and HT samples based on PC2.

#### 2.9.3. Hierarchical Clustering

**Figure 8:**
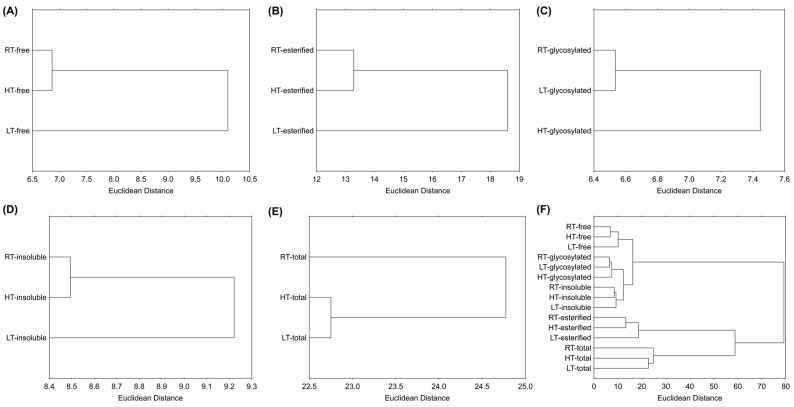
Hierarchical clustering of RT-, LT- and HT-grown broccoli microgreens (**A**) free, (**B**) esterified, (**C**) glycosylated, (**D**) insoluble-bound, (**E**) sum of all the structural forms, and (**F**) all the analyzed structural forms expressed as the Euclidean distance based on the measured phytochemicals and antioxidant capacity. RT = room temperature, LT = low temperature, HT = high temperature.

Hierarchical clustering is a technique that groups similar objects into groups called clusters [76]. It organizes similar data points into clusters, where each cluster differs from each other, while the cases inside one cluster are similar to each other. The output of this approach is a dendrogram, which provides a straightforward visual representation of the relationships within the data. According to the results, the LT group was separated from the RT and HT based on free forms (Figure 8A), esterified forms (Figure 8B), and insoluble-bound forms (Figure 8D). The HT group was separated from the RT and LT groups based on glycosylated forms (Figure 8C), and the RT group was separated from the stressed plants based on the sum of all structural forms (Figure 8E). Figure 8F shows the clustering of all the samples, where it is evident that RT, HT, and LT broccoli microgreens were the least distant from each other based on their glycosylated polyphenolic forms, while they were the most distant based on esterified forms. Additionally, samples containing esterified forms were more distant from those containing free, glycosylated, and insoluble polyphenolics, which formed a separated cluster. This suggests that the esterification of polyphenolics in broccoli microgreens is more environmentally sensitive than glycosylation or cell wall binding.

## 3. Materials and Methods

### 3.1. Plant Material

Seeds of the broccoli variety *Brassica oleracea* L. convar. *botrytis* (L.) Alef. var. *cymosa* Duch., commonly referred to as broccoli Calabrese (Art. No. 424430), were sourced from International Seeds Processing GmbH, Quedlinburg, Germany. The seeds were planted in sterile Stender B400 soil substrate and placed in a Fito-Clima 600 PLH climate chamber (Aralab, Rio de Mouro, Portugal). Plants were cultivated at room temperature (RT, 23 °C/16 h day and 18 °C/8 h dark) for 11 days, after which temperature treatments began. Three biological replicates were exposed to low temperature (LT, 12 °C/16 h day and 7 °C/8 h dark), three replicates to high temperature (HT, 38 °C/16 h day and 33 °C/8 h dark), and three replicates remained at RT as controls. Humidity was maintained at 65% across groups, except for the LT group, where it increased to 85% during the last 3 days due to the specific temperature regime. Plant material was harvested 5 days after the temperature treatments began (16 days after planting) by cutting below the lower leaves. The phenotype of plants is shown in Figure S3. The harvested material was immediately frozen under liquid nitrogen and then lyophilized in an Alpha 1-2 LSCbasic (Martin Christ, Osterode am Harz, Germany) for further analyses. All the solvents were HPLC-grade and purchased, together with all the reagents, from Merck KGaA (Darmstadt, Germany).

### 3.2. Extraction of Different Polyphenolic Structural Forms

The extraction of different polyphenolic structural forms was performed according to Arruda et al. [6] with slight modifications (Figure S4). Briefly, we extracted soluble free polyphenolics by adding 70% ethanol onto lyophilized tissue to get a final concentration of 30 mg/mL. Upon centrifugation at 5000× *g* for 5 min, the supernatant was collected, and the remaining pellet was re-extracted one more time; all supernatants were pooled and evaporated under the vacuum until the aqueous phase was obtained. The pellet was stored for the later extraction of insoluble-bound polyphenolic forms. The aqueous phase was acidified to pH 2, centrifuged (5000× *g*, 5 min), and the supernatant was extracted three times with the same volume of hexane; each time, the hexane fraction was discarded. The remaining aqueous phase was further extracted three times with the same volume of diethyl ether:ethyl acetate (1:1). Organic phases were pooled, dehydrated, and filtrated through anhydrous Na_2_SO_4_ using Whatman filter paper No. 1. Then, they were dried out under the vacuum, weighed, and dissolved in ethanol.

For the extraction of esterified polyphenolic forms, a solution containing 4 M NaOH, 10 mM EDTA, and 1% ascorbic acid was added to the aqueous phase in a 2:1 (*v*/*v*) ratio following incubation for 4 h at room temperature and 150 rpm. The pH of the obtained solution was adjusted to 2, and the solution was further extracted three times with the same volume of diethyl ether:ethyl acetate (1:1). Organic phases were pooled, dehydrated, and filtrated through anhydrous Na_2_SO_4_ using Whatman filter paper No. 1, after which they were dried out under the vacuum, weighed, and dissolved in ethanol.

For the extraction of glycosylated polyphenolic forms, 5 mL of 6 M HCl was added to the aqueous phase, and incubated for 60 min at 75 °C and 150 rpm. The solution was further extracted three times with the same volume of diethyl ether:ethyl acetate (1:1). Organic phases were pooled, dehydrated, and filtrated through anhydrous Na_2_SO_4_ using Whatman filter paper No. 1, after which they were dried out under the vacuum, weighed, and dissolved in ethanol.

Insoluble-bound polyphenolics were extracted from the pellet stored at the beginning of the extraction process. A solution containing 4 M NaOH, 10 mM EDTA, and 1% ascorbic acid was added to the pellet in a 20:1 (*v*/*w*) ratio following incubation for 4 h at room temperature and 150 rpm. The pH of the obtained solution was adjusted to 2, centrifuged (5000× *g*, 5 min), and the supernatant was re-extracted three times with the same volume of hexane. Hexane fractions were discarded, and the aqueous phase was further extracted with the same volume of diethyl ether:ethyl acetate (1:1). Organic phases were pooled, dehydrated, and filtrated through anhydrous Na_2_SO_4_ using Whatman filter paper No. 1, after which they were dried out under the vacuum, weighed, and dissolved in ethanol.

### 3.3. Spectrophotometric Analyses of Different Groups of Polyphenolics

Total phenolics were determined using Folin–Ciocâlteu reagent and 1.88 M sodium carbonate solution, according to Singleton and Rossi [77], and expressed in milligrams of gallic acid equivalents per gram of dry weight (mg GAE/g dw). Total flavonoids were assessed as in Zhishen et al. [78] using aluminum chloride, sodium nitrite, and sodium hydroxide, and expressed in milligrams of quercetin equivalents per gram of dry weight (mg QE/g dw). Tannins were determined using Folin–Ciocâlteu reagent and sodium carbonate 3.5% (*w*/*v*), as in Galvão et al. [79] and expressed in mg GAE/g dw. Total hydroxycinnamic acids and flavonols were measured using hydrochloric acid (HCl) in two different concentrations (1 g/L and 2 g/L HCl), according to Howard et al. [80] and expressed in milligrams of ferulic acid equivalents per gram of dry weight (mg FerAE/g dw) or mg QE/g dw, respectively. Total phenolic acids were measured according to Jain et al. [81] and expressed in mg CAE/g dw. All measurements were performed using FLUOstar Optima (BMG Labtech GmbH, Ortenberg, Germany).

### 3.4. High-Performance Liquid Chromatography Analysis of Individual Polyphenolic Compounds

Reversed-phase high-performance liquid chromatography (RP-HPLC) was performed according to Šola et al. [7]. Separation, identification, and quantification of individual polyphenolics were performed on an Agilent 1100 Series device with a UV/VIS detector. The separation was carried out on a Poroshell 120 SB-C18 non-polar column (4.6 × 75 mm, 2.7 μm particle size) placed behind the guard column Zorbax Rx-C18 (4.6 × 12.5 mm, 5 μm particle size). The solvents, gradient, flow-through, and determination of concentration using external standards are described in our previous work [82].

### 3.5. Determination of Antioxidant Capacity of Different Polyphenolic Structural Forms

Antioxidant capacity was measured using three standard assays; ABTS (2,2′-azino-bis(3-ethylbenzothiazoline-6-sulfonic acid)) radical scavenging assay as described in Re et al. [83], DPPH (2,2-diphenyl-1-picrylhydrazyl) radical scavenging assay according to Brand-Williams et al. [84], and FRAP (ferric ion reducing antioxidant power) assay as described in Benzie and Strain [85]. The results were expressed in the percentage of inhibition. All measurements were performed using FLUOstar Optima (BMG Labtech GmbH).

### 3.6. Statistical Analyses

The data was analyzed statistically using the Statistica 14.0 software (TIBCO Software Inc., Palo Alto, CA, USA). Pearson’s correlation coefficients were calculated to assess relationships between different structural forms of polyphenolic compounds and antioxidant capacity. To compare average values across multiple samples, one-way analysis of variance (ANOVA) and Duncan’s New Multiple Range Test (DNMRT) were employed. Differences were considered statistically significant at *p* ≤ 0.05. To evaluate the similarities or differences among samples based on their polyphenolic profile and antioxidant properties, multivariate analyses were performed, including principal component analysis (PCA), hierarchical clustering (HC) based on Euclidean distance, and single linkage clustering.

## 4. Conclusions

Structural characteristics determine the stability, bioavailability, and function of plant bioactive compounds. The structure of compounds in plants is influenced by environmental conditions. We analyzed the effects of different environmental temperatures on the structural variations of polyphenolics in broccoli microgreens. The results showed that broccoli microgreens produced more soluble polyphenolics than insoluble-bound polyphenolics, which we assume is because their tissues are softer than those of woody plants, which require higher concentrations of insoluble-bound polyphenolics for structural support. The highest proportion of insoluble-bound forms was found among tannins. Esterified forms exhibited higher antioxidant capacity than other phenolic forms. LT generally increased the amount of free and esterified forms of polyphenolics, while HT enhanced glycosylated forms, with corresponding effects on antioxidant potential. The increase in soluble over insoluble-bound polyphenolics suggests that broccoli microgreens primarily adjust to temperature stress by using these compounds as intermediates in biochemical pathways involved in maintaining redox balance, rather than in structural reinforcement. Among the individual compounds, ferulic acid emerged as a key polyphenolic in protecting broccoli against HT, while sinapic acid acted as a mediator in response to LT stress. These results provide deeper insights into how environmental factors modulate polyphenolic composition and function, ultimately affecting both plant resilience and dietary health potential.

## Figures and Tables

**Table 1:** The effect of high and low growth temperature on the concentration of free and bound ferulic and sinapic acids in broccoli.

	Ferulic Acid	Sinapic Acid
	(mg/g dw)	
free		
RT	0.28 ± 0.04 b	2.30 ± 0.41 b
HT	0.65 ± 0.06 b	2.01 ± 0.55 b
LT	0.33 ± 0.05 b	3.62 ± 0.87 ab
ΔHT (%)	135.47 *	−12.69
ΔLT (%)	17.84	57.50 *
esterified		
RT	1.36 ± 0.44 a	9.70 ± 0.25 a
HT	2.33 ± 0.48 a	9.12 ± 0.47 a
LT	1.31 ± 0.27 a	8.14 ± 4.83 a
ΔHT (%)	70.82	−6.02
ΔLT (%)	−4.03	−16.06 *
glycosylated		
RT	0.07 ± 0.01 b	0.13 ± 0.03 c
HT	0.09 ± 0.03 c	0.08 ± 0.01 c
LT	0.05 ± 0.01 b	0.07 ± 0.01 b
ΔHT (%)	36.94	−36.84
ΔLT (%)	−26.68	−46.72
bound		
RT	0.17 ± 00 b	0.99 ± 00 c
HT	0.11 ± 0.11 c	0.47 ± 0.21 c
LT	0.09 ± 0.05 b	0.62 ± 0.42 b
ΔHT (%)	−36.68	−52.21
ΔLT (%)	−49.49	−37.72
sum		
RT	1.87 ± 0.47	13.12 ± 0.51
HT	3.18 ± 0.57	11.68 ± 0.56
LT	1.77 ± 0.19	12.45 ± 3.86
ΔHT (%)	69.53 *	−10.99
ΔLT (%)	−5.68	−5.10

Different small letters denote significant differences between structural forms within the same temperature treatment, and an asterisk denotes significant differences compared to RT. RT = room temperature, HT = high temperature, LT = low temperature.

**Table 2:** The effect of high and low growth temperature on antioxidant capacity of free and bound polyphenolics in broccoli.

	ABTS (% of Inhibition)	DPPH (% of Inhibition)	FRAP (% of Reduction)
Free			
RT	48.77 ± 6.79 b	78.87 ± 2.14 a	80.90 ± 0.83 c
HT	54.93 ± 4.81 b	79.36 ± 2.32 a	80.61 ± 3.09 c
LT	59.32 ± 6.73 b	79.86 ± 1.36 a	85.46 ± 2.40 b
ΔHT (%)	12.64 *	0.63	−0.36
ΔLT (%)	21.62 *	1.26	5.63 *
Esterified			
RT	74.45 ± 3.21 a	79.59 ± 2.27 a	95.93 ± 0.63 a
HT	73.37 ± 4.75 a	78.81 ± 2.68 a	95.95 ± 0.47 a
LT	75.25 ± 3.88 a	79.43 ± 2.55 a	96.43 ± 0.45 a
ΔHT (%)	−1.45	−0.98	0.02
ΔLT (%)	1.08	−0.19	0.52 *
Glycosylated			
RT	57.71 ± 5.27 b	80.86 ± 0.97 a	86.46 ± 2.25 b
HT	64.90 ± 1.93 a	78.37 ± 1.98 a	87.75 ± 2.13 b
LT	60.35 ± 2.43 b	79.69 ± 1.27 a	86.49 ± 2.69 b
ΔHT (%)	12.45 *	−3.08 *	1.49
ΔLT (%)	4.58	−1.45	0.03
Bound			
RT	48.92 ± 21.27 b	74.18 ± 8.30 b	78.16 ± 8.03 c
HT	45.27 ± 24.89 c	79.70 ± 3.71 a	81.57 ± 5.78 c
LT	54.96 ± 10.15 b	78.29 ± 3.68 a	83.02 ± 2.62 c
ΔHT (%)	−7.47	7.44	4.37
ΔLT (%)	12.33	5.55	6.22
Sum			
RT	57.46 ± 4.97	78.37 ± 2.79	85.36 ± 1.92
HT	59.62 ± 5.58	79.06 ± 1.80	86.47 ± 2.17
LT	62.47 ± 3.68	79.32 ± 1.65	87.85 ± 1.13
ΔHT (%)	3.75	0.88	1.30
ΔLT (%)	8.71 *	1.21	2.91 *

Different small letters denote significant differences between structural forms within the same temperature treatment, and an asterisk denotes significant differences compared to RT. RT = room temperature, HT = high temperature, LT = low temperature.

**Table 3:** Pearson’s correlation coefficients between the measured variables.

Free											
	TP	TT	TPA	THCA	TF	TFLO	ABTS	DPPH	FRAP	Fer ac	Sin ac
TP	1.00										
TT	0.89	1.00									
TPA	0.98	0.96	1.00								
THCA	0.99	0.82	0.95	1.00							
TF	0.90	1.00 *	0.97	0.84	1.00						
TFLO	0.95	0.71	0.88	0.99	0.73	1.00					
ABTS	0.86	1.00 *	0.94	0.78	1.00 *	0.67	1.00				
DPPH	0.91	1.00 *	0.97	0.84	1.00	0.74	1.00	1.00			
FRAP	0.99	0.82	0.94	1.00 *	0.84	0.99	0.78	0.84	1.00		
Fer ac	−0.31	0.16	−0.12	−0.44	0.12	−0.58	0.22	0.12	−0.44	1.00	
Sin ac	0.97	0.74	0.90	0.99	0.77	1.00 *	0.70	0.77	0.99	−0.54	1.00
Esterified											
	TP	TT	TPA	THCA	TF	TFLO	ABTS	DPPH	FRAP	Fer ac	Sin ac
TP	1.00										
TT	0.95	1.00									
TPA	0.67	0.87	1.00								
THCA	1.00 *	0.94	0.64	1.00							
TF	0.63	0.84	1.00 *	0.60	1.00						
TFLO	1.00 *	0.94	0.66	1.00 *	0.62	1.00					
ABTS	0.72	0.46	−0.03	0.75	−0.08	0.73	1.00				
DPPH	0.18	−0.14	−0.61	0.22	−0.65	0.20	0.81	1.00			
FRAP	0.99	0.90	0.58	1.00	0.53	0.99	0.80	0.30	1.00		
Fer ac	−0.41	−0.09	0.41	−0.44	0.46	−0.42	−0.92	−0.97	−0.51	1.00	
Sin ac	−0.97	−0.99	−0.82	−0.97	−0.79	−0.97	−0.55	0.04	−0.94	0.19	1.00
Glycosylated											
	TP	TT	TPA	THCA	TF	TFLO	ABTS	DPPH	FRAP	Fer ac	Sin ac
TP	1.00										
TT	0.69	1.00 *									
TPA	0.77	0.99	1.00								
THCA	0.99	0.80	0.87	1.00							
TF	0.72	1.00	1.00	0.83	1.00						
TFLO	0.93	0.91	0.95	0.98	0.92	1.00					
ABTS	0.89	0.94	0.98	0.95	0.96	1.00	1.00				
DPPH	−0.83	−0.98	−1.00	−0.91	−0.98	−0.98	−0.99	1.00			
FRAP	0.99	0.77	0.84	1.00 *	0.79	0.97	0.94	−0.89	1.00		
Fer ac	0.95	0.42	0.52	0.88	0.46	0.76	0.69	−0.61	0.90	1.00	
Sin ac	−0.22	−0.86	−0.79	−0.38	−0.84	−0.56	−0.64	0.73	−0.33	0.11	1.00
Insoluble											
	TP	TT	TPA	THCA	TF	TFLO	ABTS	DPPH	FRAP	Fer ac	Sin ac
TP	1.00										
TT	−0.18	1.00									
TPA	−0.40	−0.83	1.00								
THCA	0.78	−0.76	0.26	1.00							
TF	0.62	−0.89	0.48	0.97	1.00						
TFLO	−0.53	−0.74	0.99	0.12	0.34	1.00					
ABTS	0.96	0.10	−0.64	0.57	0.37	−0.75	1.00				
DPPH	0.18	−1.00 *	0.83	0.76	0.89	0.74	−0.11	1.00			
FRAP	0.66	−0.86	0.42	0.99	1.00 *	0.29	0.42	0.86	1.00		
Fer ac	−0.63	0.88	−0.46	−0.98	−1.00 *	−0.33	−0.38	−0.88	−1.00 *	1.00	
Sin ac	−0.16	1.00 *	−0.84	−0.74	−0.87	−0.76	0.13	−1.00	−0.84	0.87	1.00
Sum											
	TP	TT	TPA	THCA	TF	TFLO	ABTS	DPPH	FRAP	Fer ac	Sin ac
TP	1.00										
TT	0.80	1.00									
TPA	0.57	0.95	1.00								
THCA	1.00 *	0.76	0.51	1.00							
TF	0.65	0.98	0.99	0.60	1.00						
TFLO	0.97	0.92	0.74	0.95	0.81	1.00					
ABTS	0.98	0.89	0.70	0.97	0.77	1.00 *	1.00				
DPPH	0.87	0.99	0.90	0.84	0.94	0.96	0.94	1.00			
FRAP	0.98	0.90	0.71	0.97	0.78	1.00 *	1.00 *	0.95	1.00		
Fer ac	−0.32	0.32	0.60	−0.38	0.51	−0.09	−0.15	0.19	−0.13	1.00	
Sin ac	−0.23	−0.77	−0.93	−0.16	−0.89	−0.45	−0.39	−0.67	−0.41	−0.85	1.00

Statistically significant correlations (*p* ≤ 0.05) are marked with an asterisk (*). TP = total phenolics, TT = total tannins, TPA = total phenolic acids, THCA = total hydroxycinnamic acids, TF = total flavonoids, TFLO = total flavonols, ABTS = 2,2′-azino-bis(3-ethylbenzothiazoline-6-sulfonic acid), DPPH = 2,2-diphenyl-1-picrylhydrazyl, FRAP = ferric ion reducing antioxidant power; Fer ac = ferulic acid; Sin ac = sinapic acid.

## Data Availability

The data that support the findings of this study are available from the corresponding author, I.Š., upon request.

## Supplementary Materials

The following supporting information can be downloaded at: https://www.mdpi.com/article/10.3390/plants14081186/s1, Figure S1: The percentage (%) of free, esterified, glycosylated, and insoluble-bound polyphenolics in the sum of all the structural forms extracted from broccoli microgreens grown under room temperature. TP = total phenolics, TT = total tannins, TPA = total phenolic acids, THCA = total hydroxycinnamic acids, TF = total flavonoids, TFLO = total flavonols; Figure S2: Intensity and direction of change of different structural forms of polyphenolic groups and their antioxidant capacity in broccoli: (A) free, (B) esterified, (C) glycosylated, (D) insoluble-bound, and (E) sum of all the structural forms. HT = high temperature, LT = low temperature, RT = room temperature, TP = total phenolics, TT = total tannins, TPA = total phenolic acids, THCA = total hydroxycinnamic acids, TF = total flavonoids, TFLO = total flavonols, ABTS = 2,2′-azino-bis(3-ethylbenzothiazoline-6-sulfonic acid), DPPH = 2,2-diphenyl-1-picrylhydrazyl, FRAP = ferric ion reducing antioxidant power. Asterisk denotes significant change compared to RT, on the level of *p* ≤ 0.05 (one-way ANOVA, Duncan’s test); Figure S3: The phenotype of plants; Figure S4: Schematic representation of the extraction process (modified according to Arruda et al. [6]).

## Abbreviations

The following abbreviations are used in this manuscript:

ABTS	2,2′-azino-bis(3-ethylbenzothiazoline-6-sulfonic acid)
CAE	caffeic acid equivalents
DPPH	2,2-diphenyl-1-picrylhydrazyl
Fer ac	ferulic acid
FRAP	ferric ion reducing antioxidant power
GAE	gallic acid equivalents
HC	hierarchical clustering
HT	high temperature
LT	low temperature
QE	quercetin equivalents
PCA	principal component analysis
RT	room temperature
Sin ac	sinapic acid
TF	total flavonoids
TFLO	total flavonols
THCA	total hydroxycinnamic acids
TP	total phenolics
TPA	total phenolic acids
TT	total tannins
