# Supplementary figures and images for: Structural Variations of Broccoli Polyphenolics and Their Antioxidant Capacity as a Function of Growing Temperature
Source: Plants (Basel). 2025 Apr 11;14(8):1186. doi: 10.3390/plants14081186 (PMC12030137; doi:10.3390/plants14081186)

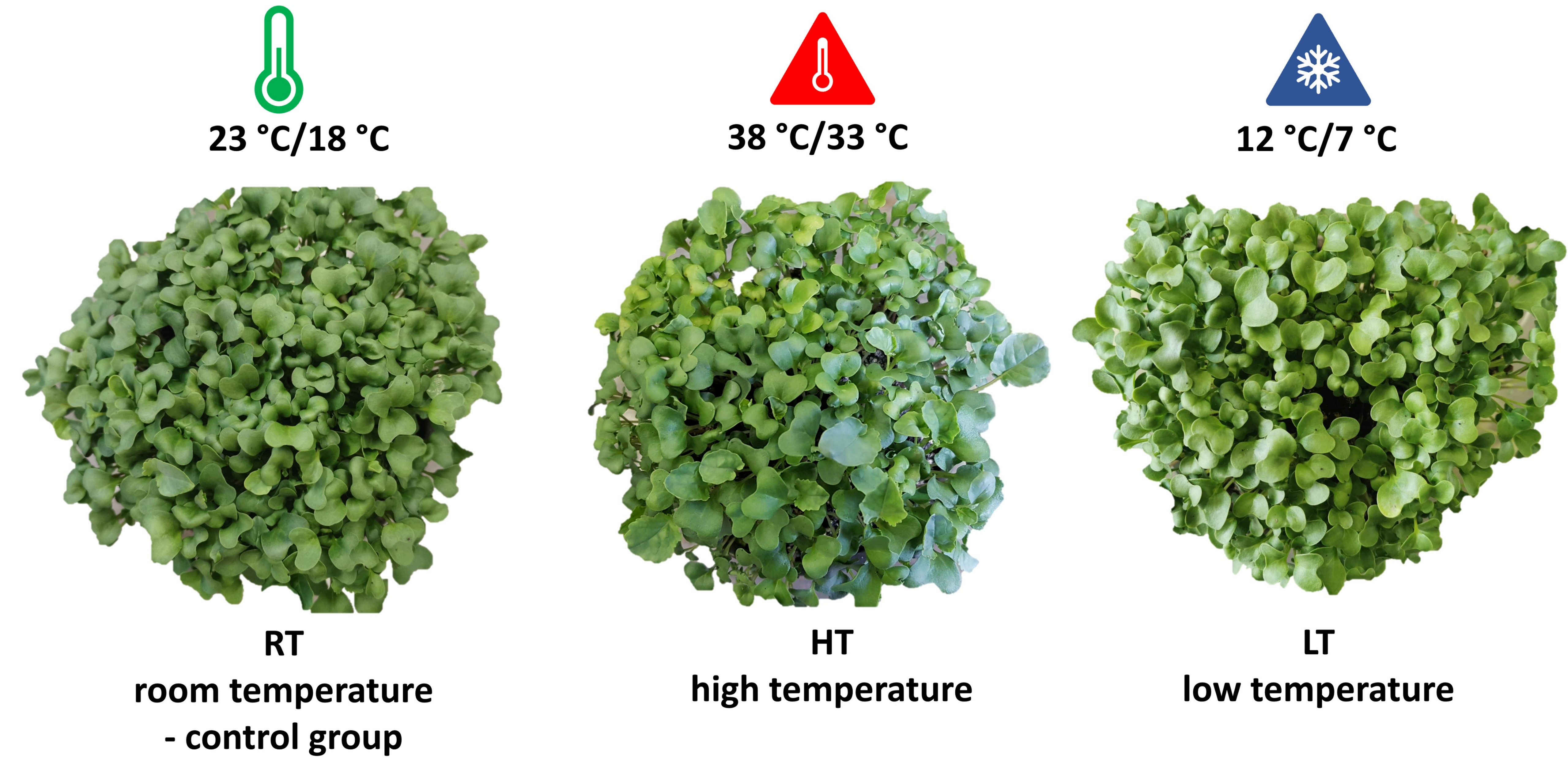

Supplement: Supplementary file 1 [file plants-14-01186-s001.zip › Figure S3.jpg]

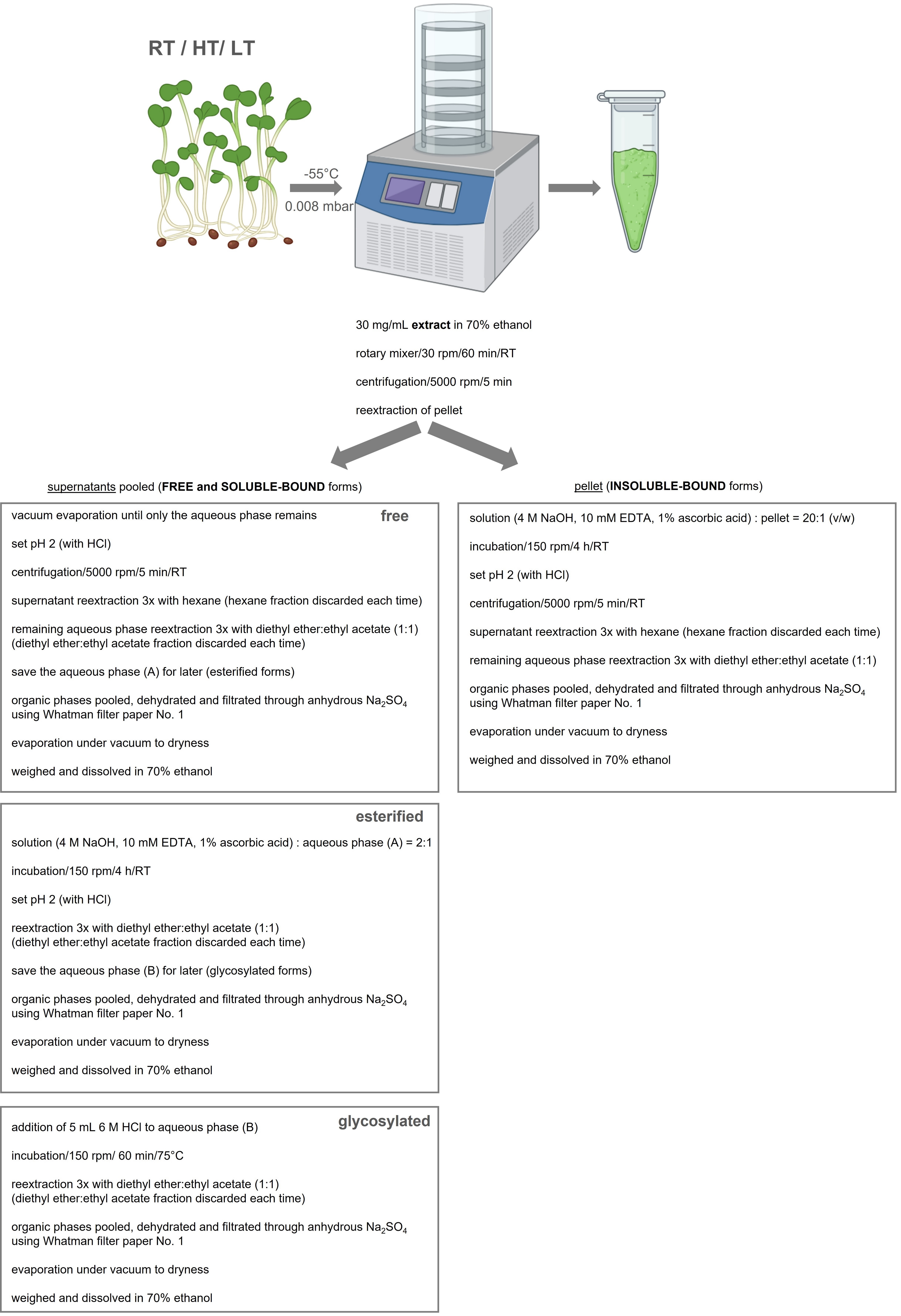

Supplement: Supplementary file 1 [file plants-14-01186-s001.zip › Figure S4.jpg]

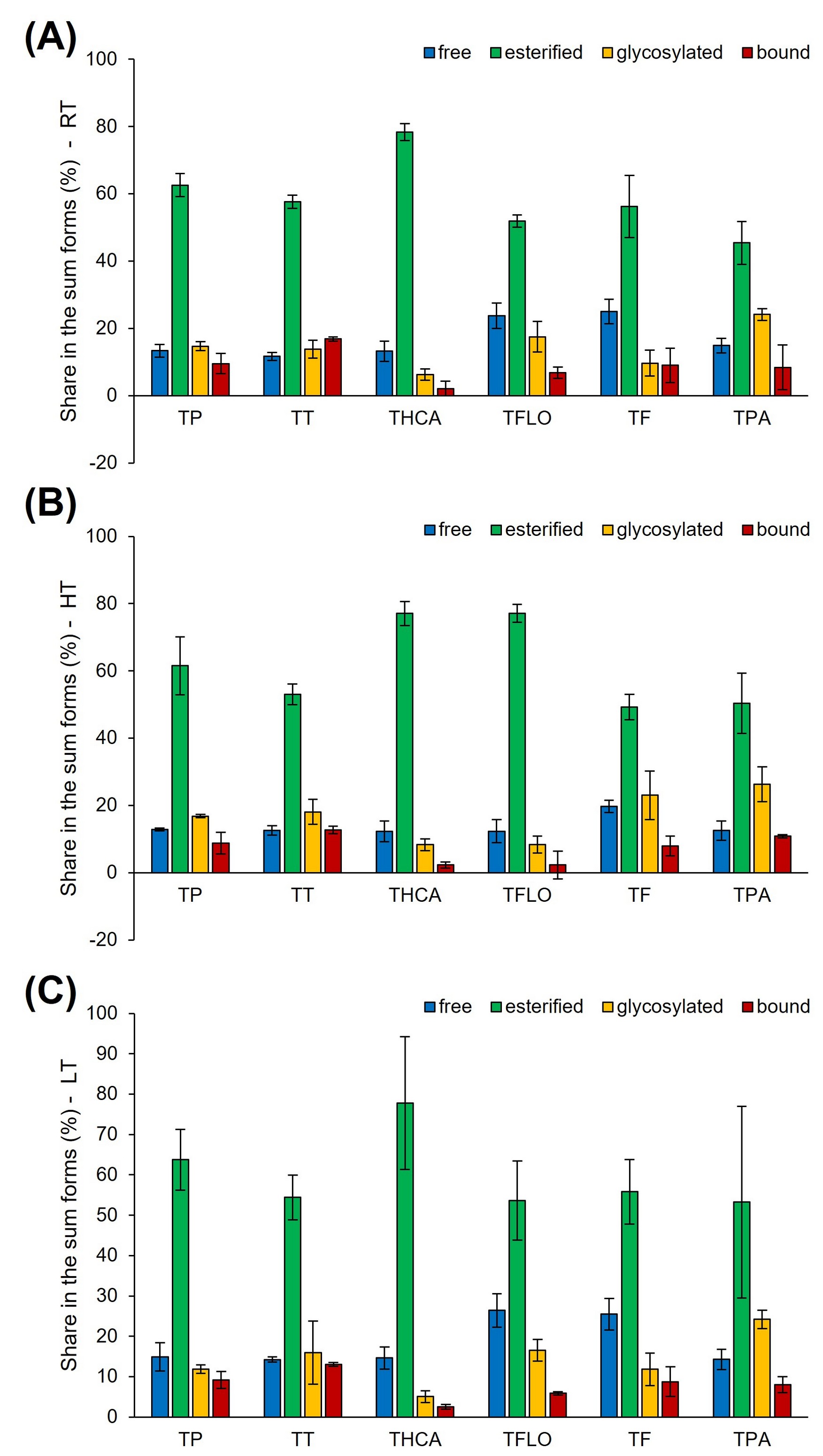

Supplement: Supplementary file 1 [file plants-14-01186-s001.zip › Figure S1.jpg]

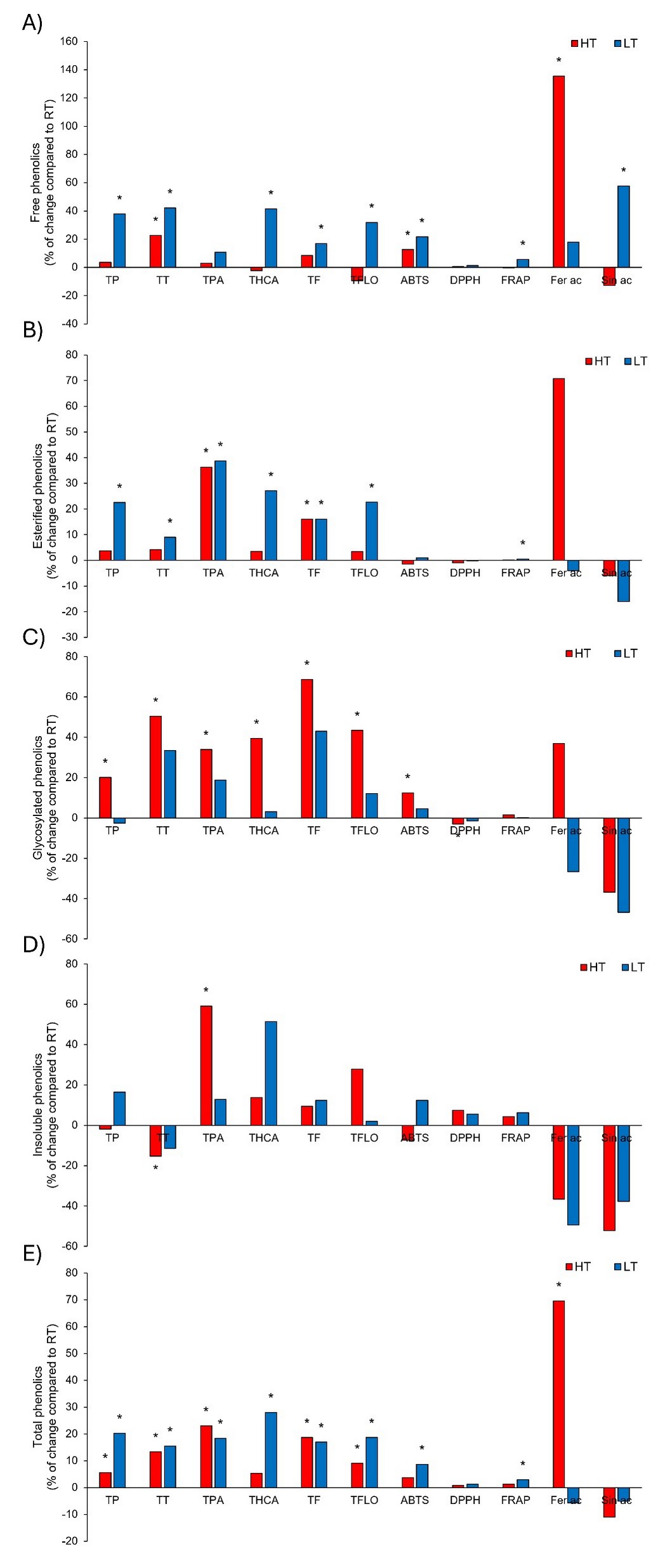

Supplement: Supplementary file 1 [file plants-14-01186-s001.zip › Figure S2.jpg]
